# Prevention of Tumor Growth and Dissemination by In Situ Vaccination with Mitochondria‐Targeted Atovaquone

**DOI:** 10.1002/advs.202101267

**Published:** 2022-03-04

**Authors:** Mofei Huang, Donghai Xiong, Jing Pan, Qi Zhang, Yian Wang, Charles R. Myers, Bryon D. Johnson, Micael Hardy, Balaraman Kalyanaraman, Ming You

**Affiliations:** ^1^ Center for Cancer Prevention Houston Methodist Research Institute 6670 Bertner Ave Houston TX 77030 USA; ^2^ Department of Pharmacology and Toxicology Medical College of Wisconsin Milwaukee WI 53226 USA; ^3^ Department of Medicine Medical College of Wisconsin Milwaukee WI 53226 USA; ^4^ Aix Marseille Univ, CNRS ICR UMR 7273 Marseille 13013 France; ^5^ Department of Biophysics Medical College of Wisconsin 8701 Watertown Plank Road Milwaukee WI 53226 USA

**Keywords:** in situ vaccination, lung cancer, mitochondria‐targeted atovaquone, mitochondrial bioenergetics, tumor immune microenvironment

## Abstract

Atovaquone, an FDA‐approved drug for malaria, is known to inhibit mitochondrial electron transport. A recently synthesized mitochondria‐targeted atovaquone increased mitochondrial accumulation and antitumor activity in vitro. Using an in situ vaccination approach, local injection of mitochondria‐targeted atovaquone into primary tumors triggered potent T cell immune responses locally and in distant tumor sites. Mitochondria‐targeted atovaquone treatment led to significant reductions of both granulocytic myeloid‐derived suppressor cells and regulatory T cells in the tumor microenvironment. Mitochondria‐targeted atovaquone treatment blocks the expression of genes involved in oxidative phosphorylation and glycolysis in granulocytic‐myeloid‐derived suppressor cells and regulatory T cells, which may lead to death of granulocytic‐myeloid‐derived suppressor cells and regulatory T cells. Mitochondria‐targeted atovaquone inhibits expression of genes for mitochondrial complex components, oxidative phosphorylation, and glycolysis in both granulocytic‐myeloid‐derived suppressor cells and regulatory T cells. The resulting decreases in intratumoral granulocytic‐myeloid‐derived suppressor cells and regulatory T cells could facilitate the observed increase in tumor‐infiltrating CD4^+^ T cells. Mitochondria‐targeted atovaquone also improves the anti‐tumor activity of PD‐1 blockade immunotherapy. The results implicate granulocytic‐myeloid‐derived suppressor cells and regulatory T cells as novel targets of mitochondria‐targeted atovaquone that facilitate its antitumor efficacy.

## Graphical abstract

Mitochondria‐targeted atovaquone (Mito‐ATO) in situ vaccination eliminates tumor cells by activating systemic CD4^+^ T response through blocking both granulocytic‐myeloid‐derived suppressor cells (G‐MDSCs) and regulatory T cells (Tregs). Mito‐ATO inhibited complex I and glycolysis in G‐MDSCs and complex III in Tregs. Targeting G‐MDSCs and Tregs with Mito‐ATO is a promising approach to eradicate disseminated tumors.

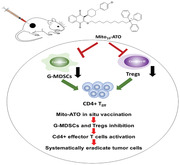

## 1 Introduction

Atovaquone (ATO) is an FDA‐approved drug for use in combination with proguanil for the prevention and treatment of malaria; it is also an FDA‐approved alternative for the prevention or treatment of *Pneumocystis* pneumonia, and an alternative for treating *Toxoplasma* in combination with sulfadiazine.^[^ ^1^, ^2^ ^]^ ATO is highly lipophilic, with limited solubility in water; its bioavailability is dependent on its formulation and diet, and its absorption is enhanced by high‐fat foods.^[^ ^2^, ^3^ ^]^ ATO is a quinone that inhibits the mitochondrial electron transport chain in *Plasmodium falciparum* at the cytochrome *bc*_1_ complex (complex III).^[^ ^4^ ^]^ ATO has been shown to have anticancer effects in multiple preclinical cancer models,^[^ ^5^ ^]^ although most of the studies with ATO have focused on its direct effects on cancer cells.^[^ ^1^, ^6^ ^]^ It remains to be determined whether ATO also exhibits tumor‐extrinsic antitumor capabilities by perturbing immune cell recognition and/or altering the host's tumor immune microenvironment (TIME).

Ample evidence supports a significant role for mitochondrial metabolism in promoting cancer development and progression.^[^ ^7^ ^]^ Conjugating delocalized lipophilic cations such as the triphenylphosphonium cation (TPP^+^) to compounds of interest is an effective approach for mitochondrial targeting.^[^ ^7a^ ^]^ The hyperpolarized potential of tumor cells and mitochondrial membranes facilitates selective accumulation of TPP^+^ conjugates in the mitochondria of various cells (including immune cells) in the TIME versus those in cells that are not associated with the TIME.^[^ ^7a^ ^]^ We therefore developed a mitochondria‐targeted ATO (Mito‐ATO) by attaching the bulky TPP^+^ group to ATO via a long alkyl chain, which separates TPP^+^ from ATO's structure and increases its lipophilicity and mitochondrial uptake in cancer cells^[^ ^1^ ^]^ and could increase its uptake in immunosuppressive cells in the TIME.

The current study explores the effects of Mito‐ATO on immune cells within the TIME in mouse tumor models. Using an in situ vaccination approach, Mito‐ATO was injected locally into tumors, which triggered a potent T cell immune response that attacked the tumor locally and subsequently attacked tumors throughout the body in both transplanted tumor models and a spontaneous tumor model. Flow cytometry analysis found that Mito‐ATO treatment decreased intratumoral (i.t.) granulocytic‐myeloid‐derived suppressor cells (G‐MDSCs) and regulatory T cells (Tregs) and increased effector CD4^+^ T cells. Single‐cell RNA sequencing (scRNA‐seq) analyses showed that the reduction of G‐MDSCs and Tregs was linked to Mito‐ATO's inhibition of oxidative phosphorylation (OXPHOS) by suppression of mitochondrial complexes and glycolysis in G‐MDSCs and Tregs, leading to the death of these cells. Thus, in situ vaccination with Mito‐ATO can decrease both myeloid‐derived suppressor cells (MDSCs) and Tregs within tumors to effectively prevent and treat local and metastatic disease.

## 2 Results

### 2.1 In Situ Treatment with Mito‐ATO Induces T Cell Immune Responses that Inhibit Tumor Growth in Both Syngeneic Mouse Models and in Animals Genetically Prone to Spontaneous Breast Cancers

**Figure 1:**
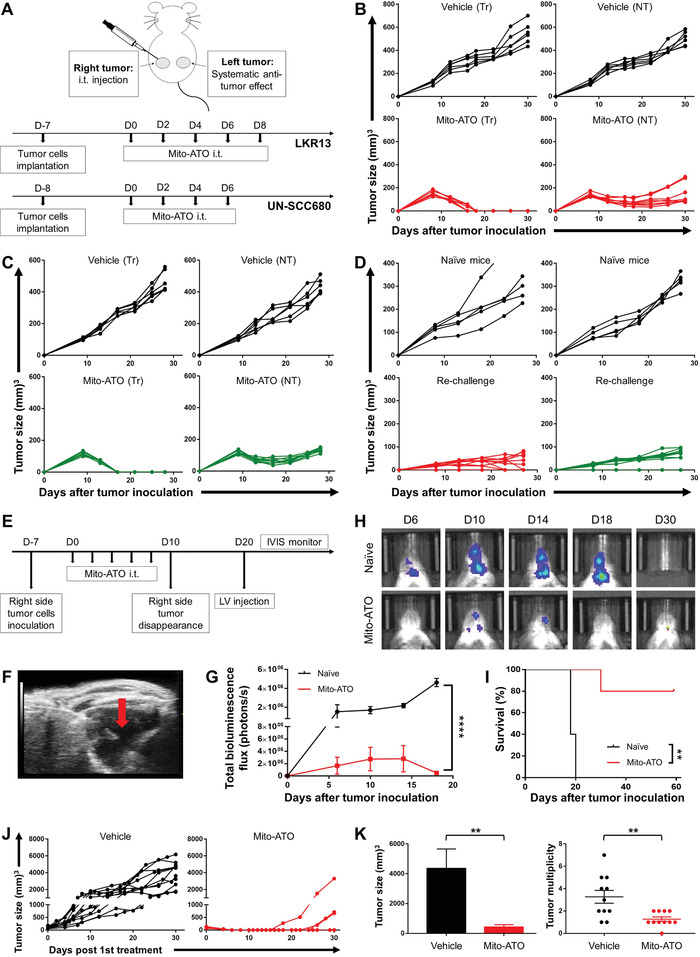
Intratumoral injection of Mito‐ATO eradicates established local tumors and induces systematic antitumor effects in various tumor models. A) Experimental design outlining the timing of tumor cell inoculation and administration of Mito‐ATO. Female SV129 or A/J mice were inoculated with LKR13 lung adenocarcinoma cells (2 × 10^6^) or UN‐SCC680 (5 × 10^6^) lung squamous cells on both the left and right sides of the abdomen. Treatment was initiated when tumors reached at 5–8 mm in diameter (usually between seven and nine days after tumor inoculation). Mice received i.t. treatment of vehicle (*n* = 6) or Mito‐ATO (*n* = 8) on the right‐side tumor every other day for a total of four or five injections. Tumor sizes on both the treated and nontreated sides were monitored by caliper every four days throughout study. B) Tumor growth of LKR13 two‐tumor model. Left column: treated‐side (Tr) tumors; right column: nontreated side (NT) tumors. C) Tumor growth of UN‐SSC680 two‐tumor model. D) In situ vaccination with Mito‐ATO prevents distant site tumor rechallenge. Left column: LKR13 tumor model; right column: UN‐SCC680 tumor model. SV129 (*n* = 10) who have rejected a primary tumor after Mito‐ATO i.t. injections were rechallenged eight days later with LKR13 lung adenocarcinoma cells (1 × 10^6^) on the opposite side abdomen, respectively. As a control, age‐matched naïve littermates (*n* = 5) were used for each strain. A/J mice (*n* = 10) received a similar strategy and were rechallenged with UN‐SCC680 lung squamous cancer cells (2 × 10^6^). Tumor sizes were monitored every four or five days at the site of rechallenge. Sizes of tumors at the site of rechallenge are shown. E) Timeline for brain metastases model development. F) High‐resolution echocardiography to visualize the position of the needle during intracardiac injection of lung cancer cells into the circulation. The red arrow indicates the injection site. G) Quantitative data for the bioluminescence imaging of brain metastases (*n* = 5). Statistical significance was calculated using two‐tailed Student's *t*‐test. H) Representative bioluminescence images of brain tumor burden in one naïve mice and one cured mice were accessed over time. I) Survival analysis of different groups of SV129 mice receiving the brain metastases challenge (*n* = 5). Statistical significance was calculated using log‐rank test. J) Tumor growth curves of tumor bearing mice treated with vehicle (*n* = 11) or Mito‐ATO (*n* = 11). K) Mito‐ATO i.t. treatment decreased tumor load (left) and tumor multiplicity (right) of nontreated tumors. Statistical significance was calculated using two‐tailed Student's *t*‐test. Data are shown as the mean ± SE, ∗ *P* < 0.05, ∗∗ *P* < 0.01, ∗∗∗ *P* < 0.001, and ∗∗∗∗ *P* < 0.0001, two‐tailed Student's *t*‐test.

To determine if Mito‐ATO could induce antitumor immune responses locally and boost systemic antitumor immunity, we implanted mice with LKR13 mouse lung adenocarcinoma cells at two different sites in the body, allowed the tumors to become established, and then injected Mito‐ATO into only one tumor site (**Figure** **1**A). Tumor growth was monitored at both the injection site and the distant site. The tumors of vehicle‐treated mice grew progressively at both sites (Figure 1B,C). Mito‐ATO caused complete tumor regression at the local injection site (*P* < 0.0001) and caused a significant delay in tumor growth at the distant site (>70% decrease; *P* < 0.0001) (Figure 1B). Mito‐ATO was also effective against UN‐SCC680 mouse lung squamous cell carcinoma when tested in a similar manner (Figure 1C). Additionally, we tested if Mito‐ATO's induction of an antitumor immune response was sufficient to reject a tumor rechallenge. We implanted mice with LKR13 mouse lung adenocarcinoma cells or UN‐SCC680 mouse lung squamous cell carcinoma cells at one site in the body; following establishment of the tumor, it was injected with Mito‐ATO. Rejection of these primary tumors for both syngeneic tumor models (LKR13 and UN‐SCC680) was assessed following completion of the Mito‐ATO treatment (Figure 1D). Then, a second tumor was established in each animal at another site; rejection of this tumor rechallenge was defined as the lack of progressive growth of implanted tumor cells for as long as the animals were observed. Compared to naïve control mice, animals that previously harbored primary LKR13 or UN‐SCC680 tumors that had been treated with Mito‐ATO rejected the secondary subcutaneous tumors by 89% (LKR13; *P* < 0.0001) and 79% (UN‐SCC680; *P* < 0.0001), even though the secondary tumors had not been injected with Mito‐ATO (Figure 1D). To investigate the potential of in situ vaccination with Mito‐ATO to prevent brain metastases, we used six‐week‐old female SV129 mice previously cured of LKR13 lung carcinoma via i.t. injection with Mito‐ATO as the hosts for generating brain metastasis (Figure 1E). We generated luciferase‐expressing variants of LKR13 lung carcinoma cells and confirmed that they efficiently formed brain metastases in control mice. An ultrasound‐guided procedure was used to secure the precise injection of lung cancer cells into the left cardiac ventricle of sv129 mice (Figure 1F). These injected lung cancer cells rapidly colonized the cerebrum and cerebellum, forming many nodules in animals treated with vehicle control (Figure 1H). Ten days after the initial subcutaneous LKR13 lung carcinoma had been cured with i.t. injection with Mito‐ATO, 5 × 10^5^ LKR13‐Luc tumor cells in 100 µL of phosphate‐buffered saline (PBS) were injected into the left ventricle of the cured mice and their age‐matched littermates under ultrasound guidance to generate brain metastases (Figure 1F). Brain metastases were monitored periodically by bioluminescence. The mice that had been previously cured by Mito‐ATO vaccination showed dramatically decreased bioluminescence signals compared with naïve control mice, with signals starting to decrease at day 14 postinoculation (Figure 1G,H). The cured mice also demonstrated a longer survival advantage, whereas all naïve mice died before or at day 20 (Figure 1I). These results suggest that the initial in situ Mito‐ATO vaccination may have elicited antitumor immune changes that are durable over time to prevent and treat cancers and their distant metastases.

To investigate the antitumor effect of in situ treatment with Mito‐ATO in a spontaneous tumor model, we used the C3(1)/Tag transgenic mouse mammary tumor model, which resembles human basal‐like triple‐negative breast cancer.^[^ ^8^, ^9^ ^]^ At about 12 weeks of age, these FVB/N background mice develop mammary intraepithelial neoplasia (sharing similarities with human ductal carcinoma in situ, a preinvasive type of breast cancer), which progress into highly invasive carcinoma at about 16 weeks of age in 100% of female mice. Multiple palpable tumors develop in various mammary glands in mice over 20 weeks of age.^[^ ^8^, ^9^ ^]^ This model allows for testing the effect of Mito‐ATO on the first palpable tumor as well as its potential to prevent the development of noninjected (nontreated) carcinomas in other mammary glands. When the first tumor reached an average size of 80 mm^3^, Mito‐ATO was injected into this initial tumor, which led to eradication of the primary tumor in 8 of the 11 mice (Figure 1J). Remarkably, mice given Mito‐ATO into the primary tumor were protected against the occurrence of independently arising tumors in other mammary glands (Figure 1K). Overall, Mito‐ATO treatment significantly reduced the tumor burden by ≈90% (*P* < 0.01) and tumor multiplicity by 60% (*P* < 0.01) compared with the control group. These results establish that localized injection of Mito‐ATO is an effective therapy for established tumors and can also provide prophylaxis for other disseminating tumors. The antitumor response that was elicited against other tumors that would otherwise have developed independently at distant sites suggests a durable response that is not limited to direct cytotoxicity of Mito‐ATO injection into the initial tumor.

### 2.2 Mito‐ATO Treatment Increased Tumor Infiltrating CD4^+^ T Cells and Reduced G‐MDSCs and Tregs in Tumors

**Figure 2:**
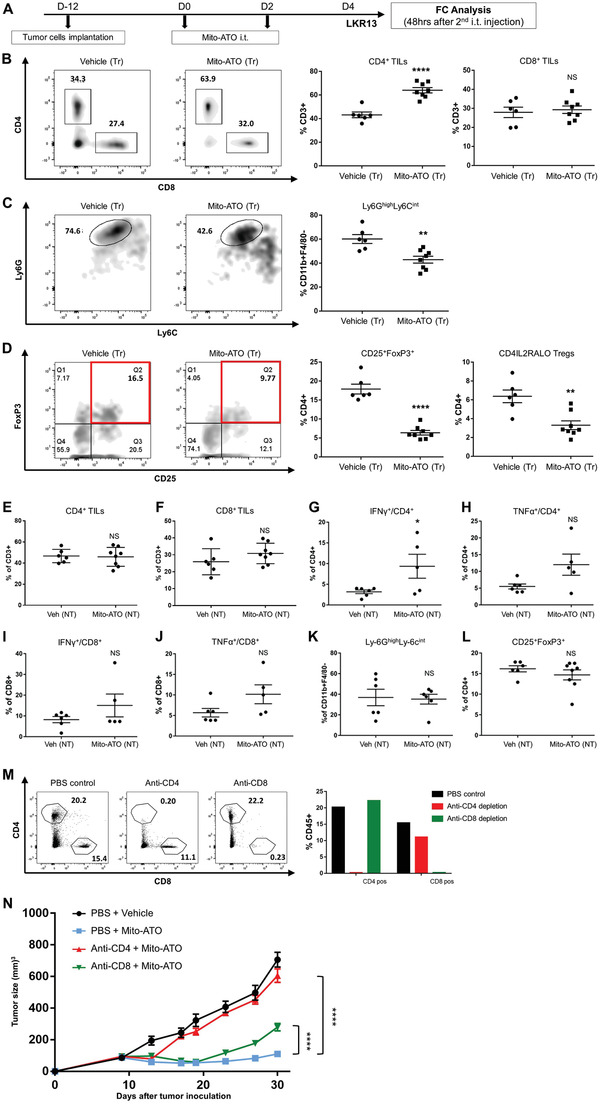
Mito‐ATO reshapes the tumor microenvironment by decreasing immune suppressive G‐MDSC and Tregs, leading to increased antitumor T cell immunity. A) KRAS‐driven LKR13 mouse lung cancer cells (2 × 10^6^) were inoculated onto both the left and right sides of the abdomens of female SV129 mice (*n* = 5–8), and Mito‐ATO i.t. treatment initiated when tumors reached around 200 mm^3^ (usually 12 days after tumor implantation). Forty‐eight hours after two Mito‐ATO injections, tumors on both sides were collected and subjected for flow cytometric analysis. B) Representative flow cytometric histograms and percentages of CD4^+^ and CD8^+^ T cells in the TIME of treated side tumors. C) Representative flow cytometric histograms and percentages of MDSCs in the TIME of treated side tumors. D) Representative flow cytometric histograms and percentages of Tregs in the TIME of treated side tumor. E) Percentage of CD4+ T cells of the nontreated side tumors. F) Percentage of CD8+ T cells of the nontreated side tumors. G) IFN‐*γ* expression in CD4+ T cells of the nontreated side tumors. H) TNF‐*α* expression in CD4+ T cells of nontreated side tumor. I) IFN‐*γ* expression in CD8+ T cells of nontreated side tumors. J) TNF‐*α* expression in CD8+ T cells of nontreated side tumor. K) Percentages of G‐MDSCs of nontreated side tumors. L) Tregs as a percent of total CD4+ T cells of nontreated side tumor. Statistical significance was calculated using two‐tailed Student's *t*‐test. M) Successful depletion was verified by flow cytometry analysis using splenocytes collected eight days after the first i.p. injection. N) Tumor growth curves of LKR13 two‐tumor model under the context of CD4^+^ or CD8^+^ T cell depletion (*n* = 6–7). Antibodies were i.p. injected on one day before and one day after tumor inoculation and repeated once per week. Statistical significance was calculated using two‐way analysis of variance. Data are shown as the mean ± SE, ∗ *P* <0.05, ∗∗ *P* < 0.01, ∗∗∗ *P* < 0.001, and ∗∗∗∗ *P* < 0.0001.

We next asked if the tumor regression benefits observed in Mito‐ATO treated mice reflect activation of tumor‐specific immune responses. We therefore conducted multicolor flow cytometric analyses of lymphocytes from the TIME of mice with implanted LKR13 lung adenocarcinomas (**Figure** **2**A). We observed that Mito‐ATO significantly increased tumor infiltrating CD4^+^ T cells (Figure 2B), and also resulted in a significant reduction of G‐MDSCs and Foxp3^+^ Tregs within tumors receiving Mito‐ATO treatment (Figure 2C,D). Interestingly, we also noted an increase in production of the cytotoxic cytokines interferon gamma (IFN‐*γ*) and tumor necrosis factor alpha (TNF‐*α*) by CD4^+^ T cells, but no changes in the total CD4^+^ or CD8^+^ T cells or G‐MDSCs or Tregs percentages in the TIME of the nontreated side tumors were evident (Figure 2E–L). To determine whether the presence of CD4^+^ or CD8^+^ T cells mediates Mito‐ATO's antitumor effects, we depleted either CD4^+^ or CD8^+^ T cells by intraperitoneal injection of anti‐mouse CD4 monoclonal antibodies (mAb) or anti‐mouse CD8 mAb in SV129 mice. Using flow cytometric analysis, we verified that these antibodies depleted >99% of the respective T cell populations (Figure 2M). These mice were implanted with LKR13 mouse lung adenocarcinoma cells at two different sites in the body, and Mito‐ATO was injected into only one tumor site. While treatment with Mito‐ATO caused a regression of the nontreated side established LKR13 adenocarcinoma in mice in which T cells had not been depleted, the antitumor effect of Mito‐ATO was abolished in mice in which CD4^+^ T cells were depleted; a much smaller decrease in Mito‐ATO's effect was observed in mice that were depleted of CD8^+^ T cells (Figure 2N). Thus, the antitumor effects of Mito‐ATO appear to require the presence of CD4^+^ T cells (and to a lesser extent, the CD8^+^ T cells) (Figure 2N).

**Figure 3:**
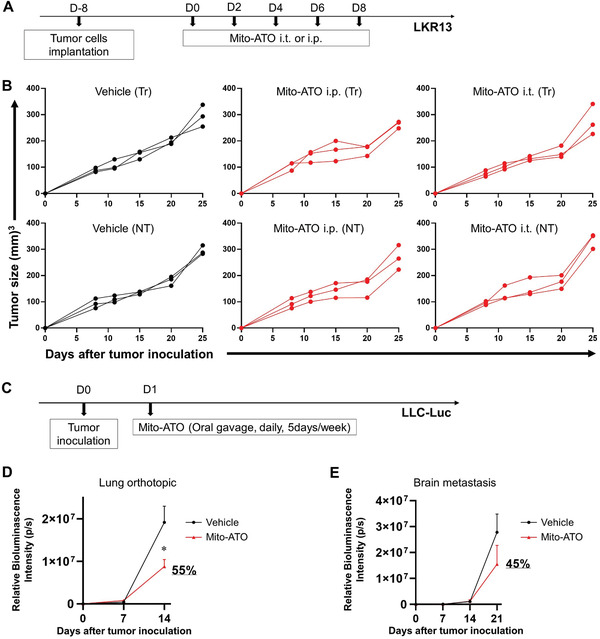
Circulating Mito‐ATO on the uninjected tumor in mice treated with Mito‐ATO in situ vaccination and effect of delivering Mito‐ATO systemically on lung tumor growth and brain metastasis. A) Experimental design outlining the timing of tumor cell inoculation and administration of Mito‐ATO. Female SV129 or A/J mice were inoculated with LKR13 lung adenocarcinoma cells (1 × 10^6^) on both the left and right sides of the abdomen. Treatment was initiated when tumors reached at 5–8 mm in diameter (eight days after tumor inoculation). B) Mice were grouped into (left panel) control group receiving both i.t. and i.p. of vehicle, (middle panel) Mito‐ATO i.p. group receiving 2 µg of Mito‐ATO through i.p. injection, and (right panel) Mito‐ATO i.t. group receiving 2 µg of Mito‐ATO through i.t. injection. Tumor sizes on both the treated and nontreated sides were monitored by caliper every four to five days throughout study. Tumor growth of LKR13 two‐tumor model. Left column: treated‐side (Tr) tumors; right column: nontreated side (NT) tumors. C–E) Delivering Mito‐ATO systemically by oral gavage inhibits tumor growth in a lung tumor orthotopic model (D) and brain metastasis of lung cancer cells using a left ventricle injection model (E). (C) Experimental timeline for delivering Mito‐ATO systematically using lung tumor orthotopic model and brain metastasis model. LLC‐Luc cells were inoculated (2 × 10^5 ^cells per mouse) inoculated either by orthotopic lung injection or left ventricle injection on D0, then one day later, oral gavage of Mito‐ATO (30 mg kg^−1^, 5 days per week) was administered. (D) Quantitative data for bioluminescence imaging of the orthotopic growth of LLC‐Luc cells (*n* = 8 per group). (E) Quantitative data for the bioluminescence imaging of brain metastases over time with Mito‐ATO treatment was started one day after injection of LLC‐Luc cells (*n* = 7). Statistical significance was calculated using two‐tailed Student's *t*‐test. Data are shown as the mean ± SE, ∗ *P* < 0.05.

To address the potential for Mito‐ATO to enter the circulation and thereby affect the noninjected tumor in treated animals, we used liquid chromatography‐mass spectrometry (LC‐MS) to detect Mito‐ATO in the circulation and in the nontreated side tumor 1 h after a single i.t. injection of 212 µg Mito‐ATO into the treated‐side tumor. We found that the amount of Mito‐ATO leaking into the circulation was around 1.8 µg, which is 0.8% of the amount we injected into the primary tumor site. Next, we examined the antitumor effect of either intraperitoneal (i.p.) or i.t. injection of 2 µg Mito‐ATO using the same LKR13 tumor model (**Figure** **3**A). No antitumor effects were observed on either the treated‐side tumor or nontreated side of mice receiving this low dose of Mito‐ATO either i.p. or i.t. as compared with the vehicle control group (Figure 3B). These data indicate that the low amounts of Mito‐ATO leaking into the circulation are insufficient to induce the observed antitumor effects of Mito‐ATO treatment on tumors not directly injected with Mito‐ATO. Thus, the antitumor effects in the nontreated side tumor appear to be mediated by a systemic T cell immune response (via circulating CD4+ T cells) triggered by i.t. Mito‐ATO injection of the treated side tumor. These data suggest that Mito‐ATO drives an adaptive T cell tumor‐specific immune response including an increase in cytotoxic CD4^+^ T cells that results in lung tumor regression; the decline in immunosuppressive G‐MDSCs and Tregs in the tumors of Mito‐ATO‐treated mice may have allowed for the expansion of CD4^+^ T cells.

We demonstrated that in situ treatment of primary tumors with Mito‐ATO generates potent systemic antitumor immunity. However, whether delivering Mito‐ATO systemically is capable of inducing antitumor activity is unknown. In a pilot study, we delivered Mito‐ATO systemically via oral gavage at a dose of 600 µg (or 30 mg kg^−1^ body weight) daily. No body weight changes were found in mice from the treated groups versus the control group. Oral Mito‐ATO administration was found to inhibit lung tumor growth by 55% and lung tumor brain metastasis by 45% using the Lewis lung carcinoma (LLC) model (injecting tumor cells either orthotopically or to the left ventricle) (Figure 3C–E).

**Figure 4:**
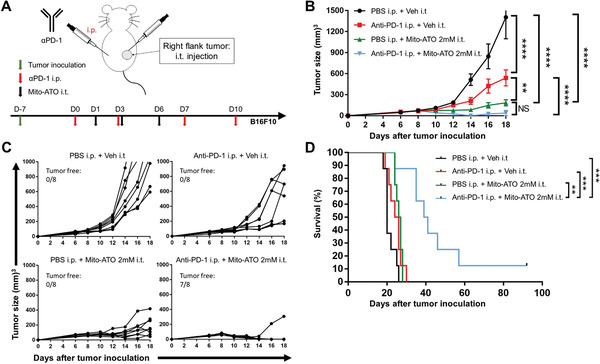
PD‐1 blockade potentiates the antitumor effect of Mito‐ATO in the B16F10 mouse melanoma model. A) Experimental design outlining the timing of B16F10 tumor cell inoculation and administration of PBS (i.p.), vehicle (i.t.), anti‐PD‐1 (i.p.), and Mito‐ATO (i.t.) in female B6 mice (*n* = 8). Tumor growth was monitored for the duration of the study. B) Average tumor size among all groups. Statistical significance was calculated using two‐way analysis of variance. C) Tumor growth dynamics in individual mouse in each experimental group. D) Survival analysis among groups. Statistical significance was calculated using log‐rank test. Data are shown as the mean ± SE, ∗ *P* < 0.05, ∗∗ *P* < 0.01, ∗∗∗ *P* < 0.001, and ∗∗∗∗ *P* < 0.0001.

Programmed cell death protein 1 (PD‐1) blockade is a part of mainstream immunotherapy in multiple malignancies; however, clinical responses are only observed in a fraction of sensitive patients. Elevated Tregs and G‐MDSCs have been demonstrated to be associated with poor outcomes of anti‐PD1 treatment.^[^ ^10^ ^]^ To improve the anticancer efficacy of anti‐PD‐1 treatment, we investigated whether combining i.t. Mito‐ATO with PD‐1 blockade could enhance its therapeutic efficacy. The B16F10 melanoma tumor model is relatively resistant to immunotherapy with anti‐PD‐1 blockade. However, mice given both i.t. Mito‐ATO (2 × 10^−3^ m dose) plus i.p. programmed death‐ligand 1 (PD‐L1) antibody treatment showed striking antitumor efficacy; in seven out of eight mice given both treatments, measurable tumors were eliminated at day 18, whereas none of mice who received anti‐PD‐1 monotherapy had their tumors cured (**Figure** **4**A–C). Furthermore, a significant survival advantage was seen in mice given Mito‐ATO plus anti‐PD‐1 compared with the control groups (Figure 4D).

### 2.3 scRNA‐seq Analyses Identify Cellular and Mitochondrial Metabolism of G‐MDSCs as a Key Target of Mito‐ATO

**Figure 5:**
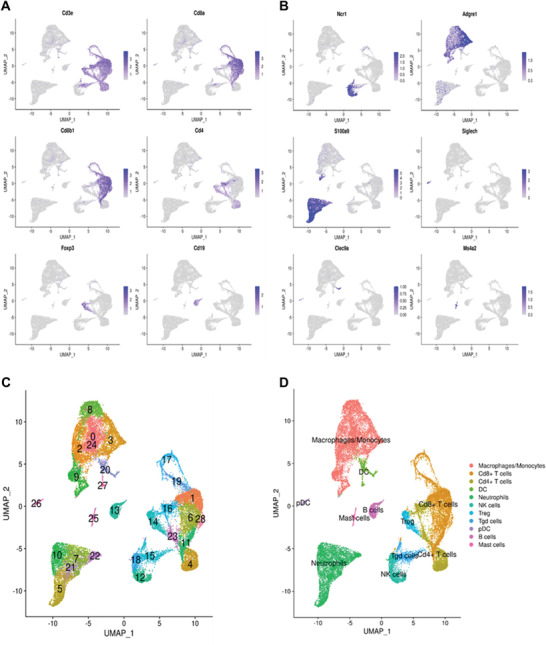
Single‐cell clustering analysis based on the full scRNA‐seq data and the annotation of each cluster based on canonical marker analysis. A) Canonical marker genes expression for T cells and B cells. B) Canonical marker genes expression for other immune cells, e.g., natural killer, macrophage, neutrophil, plasmacytoid dendritic, dendritic, and mast cells. C) Seurat‐derived clustering of all the single cells. D) The final annotation of single‐cell clusters to immune cells populations.

**Figure 6:**
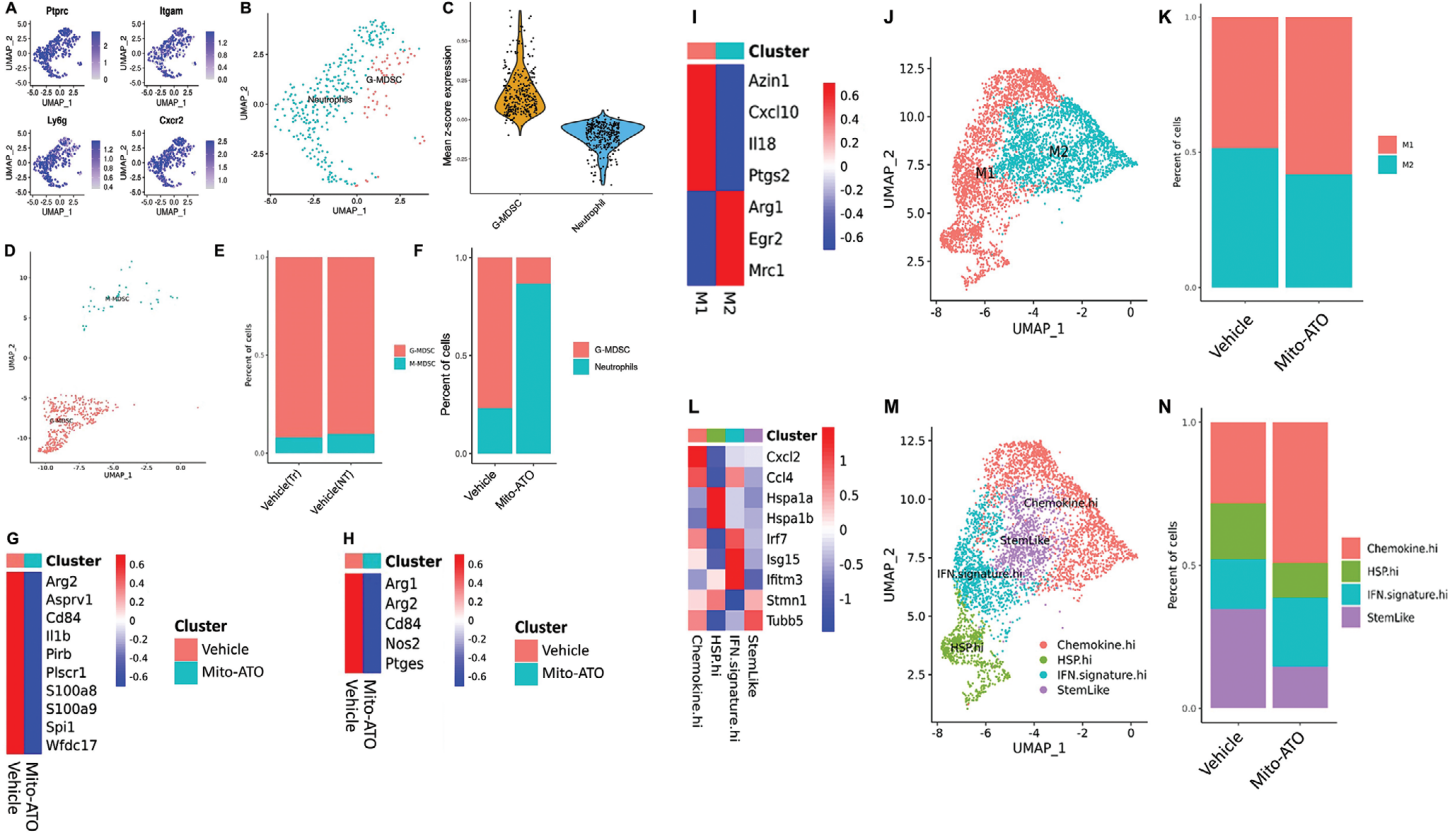
Mito‐ATO treatment reduces G‐MDSC percentage and functional genes involved in T cell suppression in mouse tumors in vivo. The inhibition of tumor G‐MDSC abundance and its glycolysis activity and the upregulation of the reactive oxygen species of tumor G‐MDSCs and apoptosis pathway activity by Mito‐ATO treatment in mice. A) General neutrophils were identified by coexpression of the following marker genes: Cd45^+^Cd11b^+^Ly6^+^Cxcr2^+^ (i.e., Ptprc^+^Itgam^+^Ly6g^+^Cxcr2^+^). B) Clustering analysis separated the two cell clusters of G‐MDSC and normal neutrophils. C) The G‐MDSC cluster showed a higher average expression of the signature genes characterized previously for the mouse G‐MDSC cells. D) UMAP plot of the MDSC cells. E) The percentages of each MDSC subtype in the treated side and untreated side mice lung tumors. F) The G‐MDSC subset percentage was drastically decreased by Mito‐ATO treatment in mouse tumors relative to the control group. G) The scRNAseq analysis showed that Mito‐ATO treatment inhibited the expression of functional genes in G‐MDSCs, including *Arg2*, *Cd84*, *Asprv1*, *Plscr1*, *Pirb*, *Wfdc17*, and *Il1b*. H) The scRNAseq analysis showed that Mito‐ATO treatment inhibited the expression of functional genes in M‐MDSCs, including *Arg1/2* and *Nos2*. I) The marker gene expression that was used to identify the M1 and M2 populations. J) Overall clustering of the macrophages into M1 and M2 subpopulations. K) Percentages of M1/M2 macrophage populations in the vehicle and Mito‐ATO treated mice lung tumors. L) Marker gene expression that was used to identify the macrophage subpopulations. M) Overall clustering of the macrophages into four subpopulations: Chemokine.hi, HSP.hi macrophages, IFN.signature.hi, and StemLike macrophages. N) Proportional changes of these four macrophage subpopulations after Mito‐ATO treatment as compared to the control group.

To identify mechanisms mediating the improved antitumor responses in mice treated with Mito‐ATO, scRNA‐seq analysis was performed on CD45+ tumor infiltrating lymphocytes (TILs) in replicate samples from each treatment (vehicle vs Mito‐ATO) using the LKR13 tumor model. The expression of the canonical markers for different types of immune cell populations were studied to annotate immune cell clusters (**Figure** **5**A,B). All cells were projected into 28 different clusters (Figure 5C), which were annotated as different types of immune cells (Figure 5D). From the total CD45^+^ immune cells, we identified the general neutrophil population as cells that coexpressed the marker genes CD45^+^, CD11b^+^, Ly6g^+^, and Cxcr2^+^ (i.e., Ptprc^+^Itgam^+^Ly6g^+^Cxcr2^+^) (**Figure** **6**A); these marker genes were established in a previous study.^[^ ^11^ ^]^ Then, we separated the G‐MDSC cell population from normal neutrophils based on their higher average expression of signature genes that were previously characterized for mouse G‐MDSC cells (Figure 6B,C).^[^ ^11^ ^]^ Using this approach, we were then able to examine the effects of Mito‐ATO treatment on G‐MDSC cells. Relative to mice treated with vehicle control, Mito‐ATO treatment markedly decreased the percentage of G‐MDSCs, which is the major type of MDSCs in this LKR13 tumor model (Figure 6D,E,F). Percentages of G‐MDSCs versus neutrophils in the tumors also demonstrated a notable decrease in Mito‐ATO treated animals (77% in the control group vs 13% in the Mito‐ATO group) (Figure 6F), further indicating that Mito‐ATO suppresses the abundance of tumor G‐MDSCs in vivo. Next, we did an sc‐RNAseq analysis of the transcripts that have been reported to be related to immunosuppressive functions of MDSCs.^[^ ^11^, ^12^ ^]^ We found Mito‐ATO treatment downregulated the G‐MDSC signature genes such as *Arg2*, *Cd84*, *Asprv1*, *Plscr1*, *Pirb*, *Wfdc17*, *Il1b*, etc., compared to the vehicle‐treated group (Figure 6G). In addition, monocytic‐myleoid‐derived suppressor cells (M‐MDSCs) in the Mito‐ATO treatment group showed downregulation of key M‐MDSC functional genes, like *Arg1/2* and *Nos2*, that are involved in T cell suppression (Figure 6H). Overall, Mito‐ATO treatment results in a significant decline in G‐MDSCs and in decreased gene expression associated with G‐MDSCs (*Arg2*, *Cd84*, *Asprv1*, *Plscr1*, *Pirb*, *Wfdc17*, *Il1b*). Because of the large decline in G‐MDSCs, the relative proportion of M‐MDSCs does increase, but decreases in M‐MDSC gene expression relevant to their ability to suppress T cells (e.g., *Arg1/2*, *Nos2*) indicate decreased M‐MDSC function. Together, the results are consistent with a decrease in overall MDSC function.

We also examined changes in other myeloid cells such as tumor associated macrophages upon Mito‐ATO treatment. There are two major macrophage populations: antitumor M1 macrophages and protumor M2 macrophages.^[^ ^13^ ^]^ The phenotype of CD11b+ F4/80+ Ly6C‐lo macrophages changed in the direction of increased percentages of antitumor M1 macrophages upon Mito‐ATO treatment (Figure 6I–K). In addition to the traditional M1/M2 macrophage analysis, we also performed deep clustering of macrophages and found a total of four macrophage subpopulations that were reported as newly identified macrophage subsets (Figure 6L).^[^ ^14^ ^]^ These macrophage subsets were termed Chemokine.hi macrophages, HSP.hi macrophages, IFN.signature.hi macrophages, and StemLike macrophages, which were defined by the overexpression of the corresponding key marker genes as previously reported.^[^ ^14^ ^]^ The clustering and proportional changes of these macrophage subpopulations are presented in Figure 6M. It was found that the Chemokine.hi and IFN.signature.hi macrophage subsets increased after Mito‐ATO treatment (Figure 6N). These two macrophage populations are similar to the macrophage population described as “inflammatory macrophages” by previous publications and had the M1 macrophage features of fostering an inflammatory response against invading tumor cells.^[^ ^14^, ^15^ ^]^ We also observed a decrease in HSP.hi macrophages and StemLike macrophages with Mito‐ATO treatment (Figure 6N). The HSP.hi macrophages belong to the Cluster 8 macrophages identified previously that had M2‐associated functions.^[^ ^14^ ^]^ The StemLike macrophages are similar to cancer stem cells with protumorigenesis properties.^[^ ^14^, ^16^ ^]^ Therefore, the percentage changes in macrophage subpopulations by Mito‐ATO treatment were favorable to the antitumor response.

**Figure 7:**
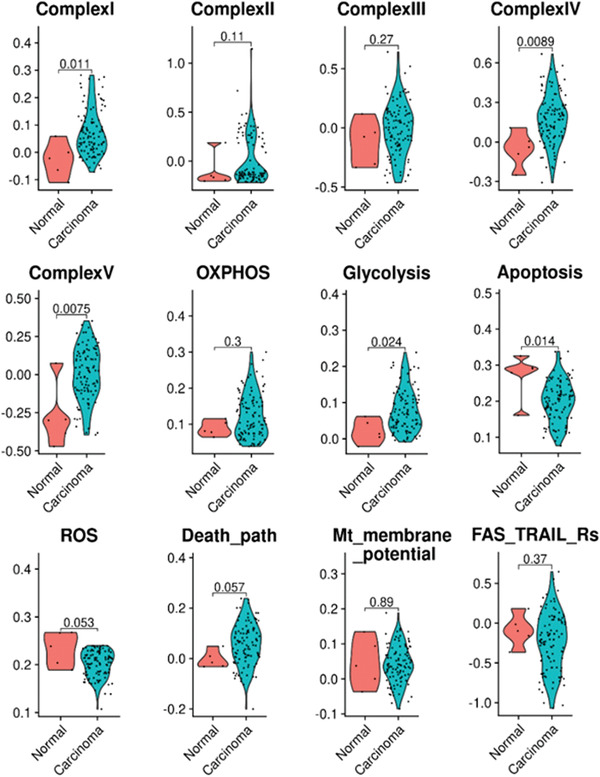
The violin plots of the key pathway activity profiles across the mouse normal esophageal tissues and the esophageal tumors. The violin plots between the normal and cancer tissues represent the changes of the pathway activity profiles of multiple mitochondrial complex assemblies and metabolic and molecular pathways that were significantly changed in the G‐MDSC cells of mouse esophageal tumors compared with the normal esophageal tissues.

**Figure 8:**
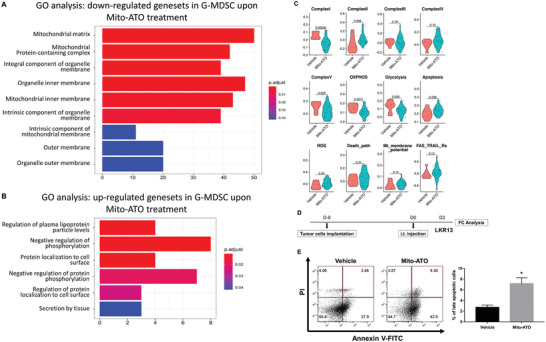
Mito‐ATO treatment altered the activity of key molecular pathways within G‐MDSC cells in mouse tumors in vivo. A) Downregulated genesets in G‐MDSCs upon Mito‐ATO treatment using Gene Ontology (GO) analysis. B) Upregulated ogenesets in G‐MDSCs upon Mito‐ATO treatment using Gene Ontology (GO) analysis. C) Mito‐ATO treatment reduced mitochondrial complex I, complex V, OXPHOS, and glycolysis activity but significantly associated with the elevated apoptosis and reactive oxygen species activity. D) Treatment timeline for testing Mito‐ATO induced G‐MDSCs using Flow cytometric assay. Seven‐week‐old female SV129 mice (*n* = 3) were inoculated with 2 × 10^6^ LKR13 cells on the right side of the abdomen. At eight days postinoculation, Mito‐ATO or vehicle was injected i.t. Forty‐eight hours later, G‐MDSCs within the tumor‐infiltrating lymphocytes were stained for apoptotic markers and subjected for flow cytometric analysis. E) Representative flow cytometric histograms and percentages of late apoptotic G‐MDSCs in the TIME. Statistical significance was calculated using two‐tailed Student's *t*‐test. Data are shown as the mean ± SE, ∗ *P* < 0.05, ∗∗ *P* < 0.01, ∗∗∗ *P* < 0.001, and ∗∗∗∗ *P* < 0.0001.

To uncover mechanisms underlying the reduced G‐MDSC percentages in TIME from mice treated with Mito‐ATO, we first determined the gene expression differences associated with mitochondrial metabolism, glycolysis, and apoptosis within G‐MDCSs in the mouse tumors relative to G‐MDSCs in normal tissues. By analyzing a publicly available scRNA‐seq dataset of mouse normal tissues and the corresponding tumors,^[^ ^17^ ^]^ we found that G‐MDSCs from tumor‐bearing mice upregulated genes for glycolysis and suppressed proapoptosis genes in G‐MDSCs in the TIME when compared with those in normal tissues (**Figure** **7**). We also did Gene Ontology (GO) analysis to have an unbiased view on the potential pathway and functional changes in G‐MDSCs upon Mito‐ATO treatment. In G‐MDSC cells, mitochondrial complex components and mitochondrial membrane genesets are the top downregulated pathways associated with Mito‐ATO treatment (**Figure** **8**A). Consequently, negative regulation of OXPHOS was enhanced in the G‐MDSC cells (Figure 8B). We did not find other major pathways significantly altered in the G‐MDSC cells. Based on these observations, we hypothesized that Mito‐ATO reduces G‐MDSCs in tumors by targeting their cellular and mitochondrial metabolism. Next, we tested the effects of Mito‐ATO on the expression of genes for key molecular pathways within the G‐MDSC cells in mouse tumors in vivo. Gene expression for mitochondrial complexes and other main energy pathways in mouse tumor G‐MDSCs were downregulated by Mito‐ATO treatment while proapoptotic genes were upregulated by Mito‐ATO treatment in tumor G‐MDSC cells (Figure 8C). Specifically, in tumor G‐MDSCs, Mito‐ATO treatment was significantly associated with reduced expression of genes for mitochondrial complex I, complex V, OXPHOS, and glycolysis, and was significantly associated with elevated gene expression for apoptosis and mitochondrial complex II (Figure 8C). The increase in genes involved in apoptosis was further supported by flow cytometry results, as we found that Mito‐ATO could induce late apoptotic cell death in G‐MDSCs in vivo (Figure 8D,E). Gene expression changes associated with these pathway activity changes are shown in **Figure** **9**. These data suggest that the inhibition on G‐MDSC cell abundance in tumors by Mito‐ATO may be mediated by suppressing the activity of key pathways of mitochondrial electron transport, OXPHOS, glycolysis, and by induction of genes for apoptosis.

Figure 9The gene expression changes that associated with the overall pathway activity changes between the control and Mito‐ATO treatment groups in mouse G‐MDSC cells. The expression of the genes of the following key pathways were downregulated in the tumor g‐MDSC cells by Mito‐ATO treatment in mice (*P* < 0.05): A) mitochondrial complex I, B) mitochondrial complex V, C) OXPHOS, and D) glycolysis. By contrast, the expression of the genes of the following key pathways were upregulated in the tumor g‐MDSC cells by Mito‐ATO treatment in mice (*P* < 0.05): E) apoptosis and F) ROS.

**Figure:**
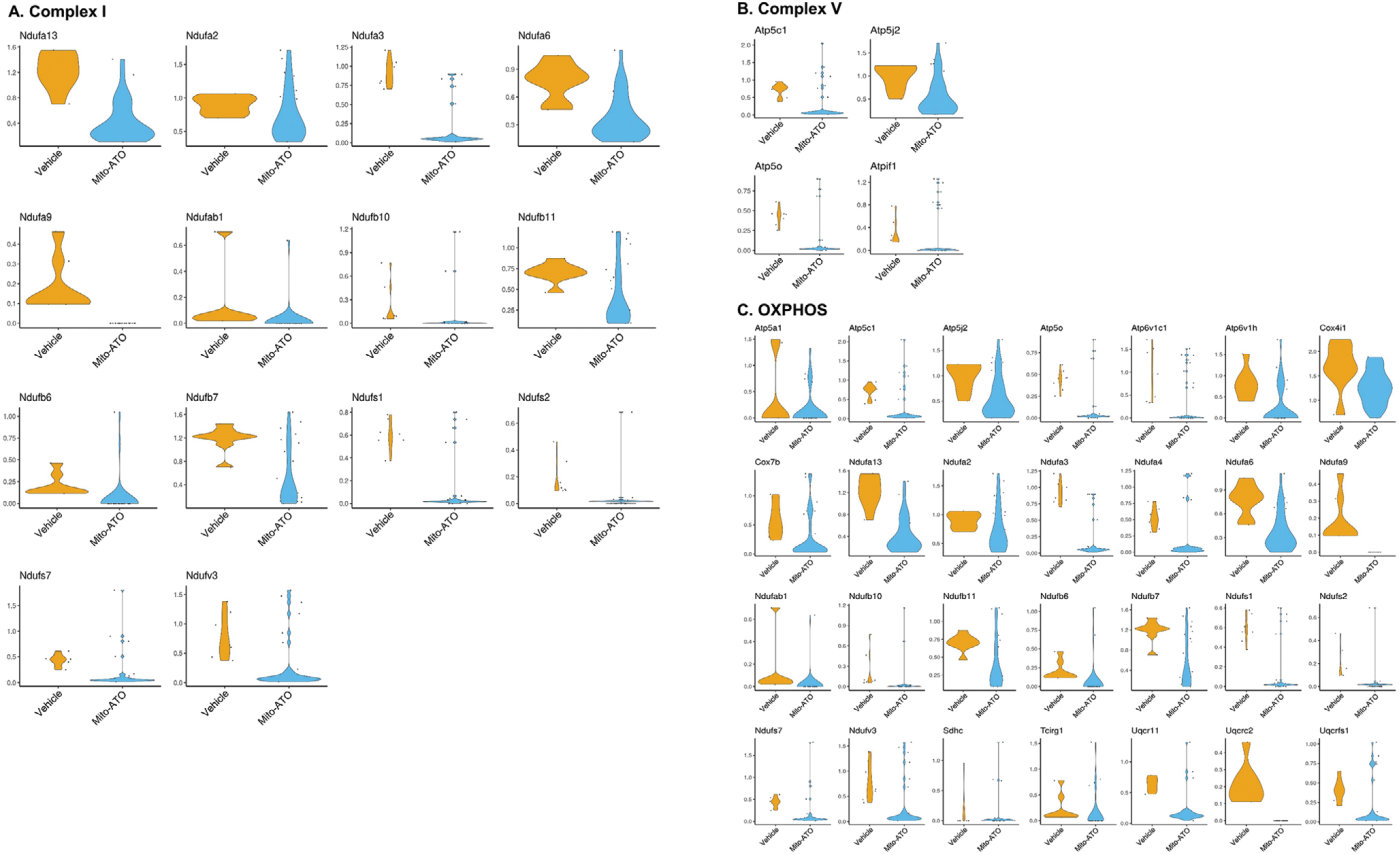

**Figure:**
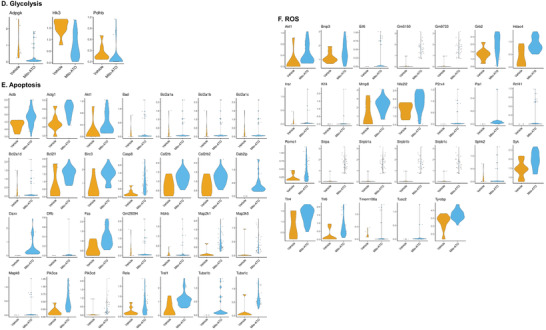

### 2.4 scRNA‐seq Analyses Reveal that Mito‐ATO Targets OXPHOS Gene Expression in a Novel Treg Subset (CD4IL2RALO T Cells)

**Figure 10:**
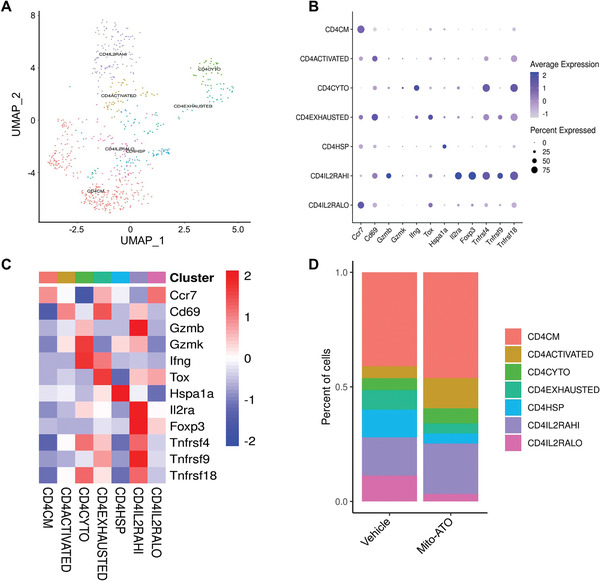
Analysis of Cd4^+^ T cells in TILs by Mito‐ATO treatment. A) Clustering analysis showed that the overall mouse CD4^+^ T cells consists of seven subpopulations. B) Dotplots of each mouse CD4^+^ T cell subpopulation expressing the indicated marker genes. C) Heatmap of each mouse CD4^+^ T cell subpopulation expressing the indicated marker genes. D) Stacked barplots of the percentages of each of the seven CD4^+^ T cells subpopulations across the control and Mito‐ATO treatment groups.

**Figure 11:**
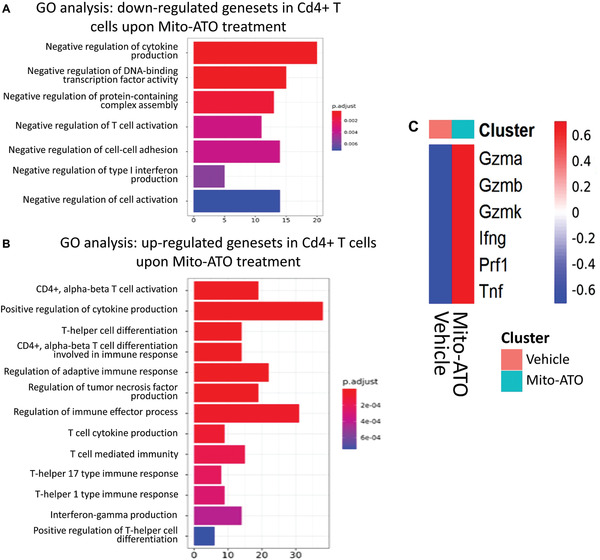
Gene Ontology (GO) analysis of CD4+ T cells (non‐Tregs) and cytotoxic cytokine analysis. A) Downregulated genesets in CD4+ T cells upon Mito‐ATO treatment. B) Upregulated ogenesets in CD4+ T cells upon Mito‐ATO treatment. C) scRNAseq analysis of cytotoxic cytokines in CD4^+^ T cells. The scRNAseq analysis showed that upon Mito‐ATO treatment CD4^+^ T cells express higher levels of cytotoxic cytokines including *Gzma, Gzmb, Gzmk, Ifng, Prf1*, and *Tnf‐alpha*.

Fine clustering analysis identified seven major CD4^+^ T cell clusters, which recapitulated the CD4^+^ T cell subclusters identified from a recent human bladder cancer study.^[^ ^18^ ^]^ The CD4^+^ T cell clusters we identified (**Figure** **10**A,B) include 1) CD4CM, central memory CD4^+^ T cells expressing *Ccr7*, 2) CD4ACTIVATED, activated CD4^+^ T cells expressing *Cd69*, 3) CD4CYTO, cytotoxic CD4^+^ T cells expressing *Gzmb*, *Gzmk*, and *Ifng*, 4) CD4EXHAUSTED, exhausted CD4^+^ T cells expressing the exhaustion‐related transcription factor *Tox*, 5) CD4HSP, inactivated CD4^+^ T cells expressing *Hspa1a*, and 6) two classes of Tregs, CD4IL2RAHI and CD4IL2RALO, that respectively had high and low expression of *Il2ra*. Both types of Tregs expressed *Foxp3*, which was not expressed in the other CD4^+^ T cell clusters. The two Treg states were differentiated by higher expression of *Il2ra*, *Tnfrsf4*, *Tnfrsf9*, and *Tnfrsf18* in CD4IL2RAHI cells, which was observed in our data (Figure 10B,C) and published data on human bladder tumors.^[^ ^18^ ^]^ Mito‐ATO treatment significantly changed the percentages of some CD4^+^ T cell clusters in the mouse tumors (Figure 10D). Specifically, the percentage of CD4ACTIVATED increased from 5% to 13%, and the percentages of CD4EXHAUSTED and CD4HSP cells decreased from 9% to 4% and from 12% to 4%, respectively, in the Mito‐ATO group compared with the control group (Figure 10D). In addition, the immunosuppresive Treg subset CD4IL2RALO decreased significantly from 10% in the control group to 3% in the Mito‐ATO group (Figure 10D). CD4^+^ T cells are thought to indirectly mediate cytotoxic effects by secreting granzyme B, IFN‐*γ*, TNF‐*α*, and IL‐2 and perform in an MHC class II‐restricted fashion.^[^ ^19^ ^]^ In the GO analysis for Cd4+ T cells (non‐Treg), the significant downregulated genesets included “negative regulation of cytokine production,” “negative regulation of type I interferon production,” “negative regulation of T cell activation,” etc. (**Figure** **11**A). The significantly up‐regulated genesets in non‐Treg Cd4+ T cells included “positive regulation of cytokine production,” “Cd4‐positive, alpha‐beta T cell activation,” “T‐helper cell differentiation,” etc. (Figure 11B). Indeed, we found CD4^+^ T cells in Mito‐ATO treated group express significantly higher levels of the activation marker *CD69*, and cytokines including *Gzma*, *Gzmb*, *Gzmk*, *Ifng*, *Prf1*, and *TNF‐α* than those in the control group (Figures  11C).

**Figure 12:**
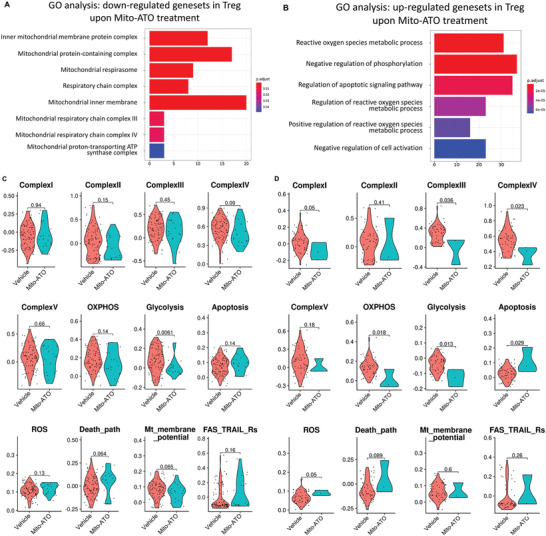
The activity profiles of multiple mitochondrial complex assemblies, metabolic and molecular pathways were significantly changed in the CD4IL2RALO Tregs by Mito‐ATO treatment. A) Downregulated genesets in Tregs upon Mito‐ATO treatment using GO analysis. B) Upregulated ogenesets in Tregs upon Mito‐ATO treatment using GO analysis. C) Comparison of mitochondrial complex and metabolic pathway activities between the control and Mito‐ATO treatment groups in the CD4IL2RAHI Tregs. D) Comparison of mitochondrial complex and metabolic pathway activities between the control and Mito‐ATO treatment groups in the CD4IL2RALO Tregs. Three types of mitochondrial complexes, i.e., Complex I, III, and IV of CD4IL2RALO Tregs, were all significantly inhibited by Mito‐ATO treatment. Two main metabolic pathways—OXPHOS and glycolysis activities—were both significantly downregulated in the Mito‐ATO group compared with the control group. Apoptosis and reactive oxygen species pathway activities were upregulated in the Mito‐ATO group.

**Figure 13:**
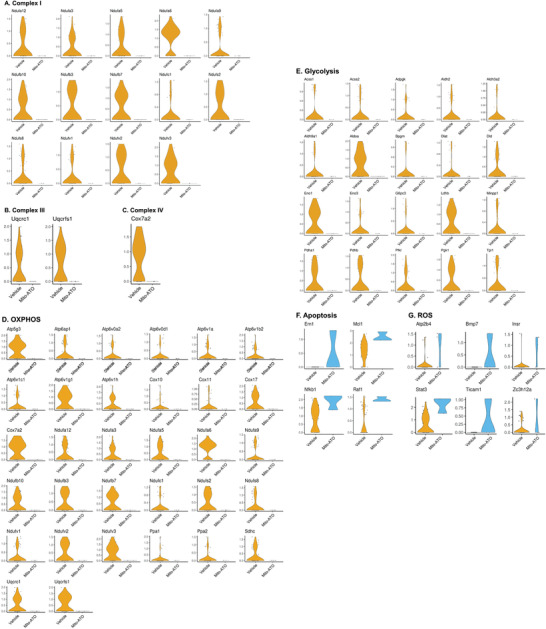
The gene expression changes associated with the overall pathway activity changes between the control and Mito‐ATO treatment groups in mouse CD4IL2RALO Tregs. The expressions of the genes of the following key pathways were downregulated in the tumor CD4IL2RALO Tregs by Mito‐ATO treatment in mice (*P* < 0.05): A) mitochondrial complex I, B) mitochondrial complex V, C) OXPHOS, and D) glycolysis. By contrast, the expression of the genes of the following key pathways were upregulated in the tumor CD4IL2RALO Tregs by Mito‐ATO treatment in mice (*P* < 0.05): E) apoptosis and F) ROS.

**Figure 14:**
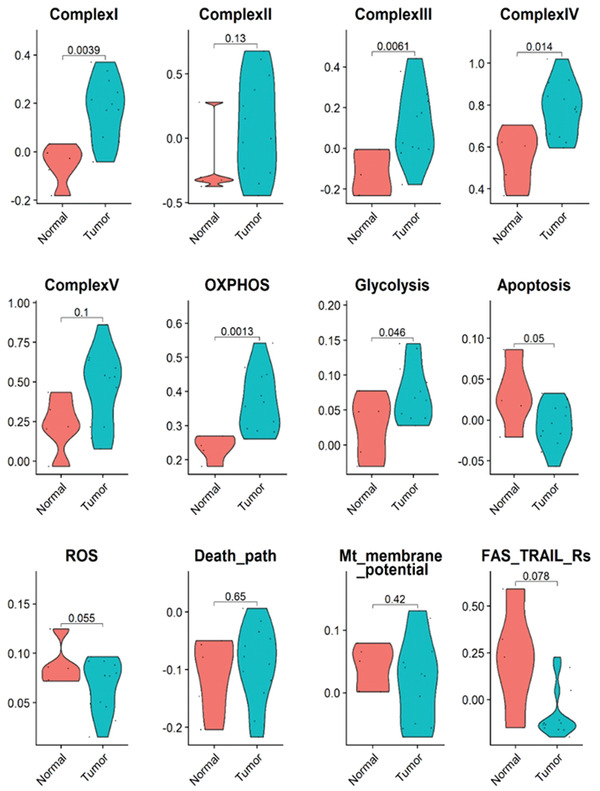
The activity profiles of multiple mitochondrial complex assemblies and metabolic and molecular pathways were significantly changed in the Tregs of mouse esophageal tumors compared with the normal esophageal tissues. The activity the of mitochondrial complexes, i.e., Complex I, III, and IV; OXPHOS pathways; and glycolysis, of the mouse tumor Tregs was significantly higher than that of the control normal Tregs. In addition, Tregs from tumor‐bearing mice had upregulated apoptosis and reactive oxygen species pathway activity compared with the CD4IL2RALO Tregs from the normal esophageal tissue samples.

Many reports have shown that mitochondrial electron transport complexes and related pathways such as OXPHOS and glycolysis are essential for the immunosuppressive function of Tregs.^[^ ^1^, ^7^, ^20^ ^]^ Interestingly, in the GO analysis for Tregs, we found mitochondrial complex components, mitochondrial membrane, and mitochondrial respirasome genesets are the top downregulated pathways (**Figure** **12**A). Correspondingly, negative regulation of OXPHOS and the apoptotic signaling pathway was enhanced in Treg cells (Figure 12B). No other pathways were found to be significantly changed in Treg cells. Therefore, we further analyzed the impact of Mito‐ATO treatment on these pathways in the two Treg states, CD4IL2RAHI and CD4IL2RALO. In the CD4IL2RAHI Tregs, only genes for glycolysis were marginally inhibited by Mito‐ATO (Figure 12C). By contrast, gene expression of multiple mitochondrial complex components and metabolic pathways were significantly decreased in the CD4IL2RALO Tregs by Mito‐ATO treatment (Figure 12D). Gene expression of three mitochondrial complexes (I, III, and IV) in CD4IL2RALO Tregs were all significantly decreased by Mito‐ATO treatment (Figure 12D). Genes for OXPHOS and glycolysis were also significantly downregulated in the Mito‐ATO group compared with the control group (Figure 12D). Coordinately, proapoptotic genes were upregulated in the Mito‐ATO group (Figure 12D). Gene expression changes associated with these pathway activity changes in CD4IL2RALO Tregs are included in **Figure** **13**. These data suggest that mitochondrial function and energy generation were severely impaired by Mito‐ATO in the CD4IL2RALO Tregs, resulting in growth inhibition of these cells and also potentially promoting increased apoptosis. Using publicly available scRNA‐seq datasets of mouse normal tissues and tumors,^[^ ^17^ ^]^we compared the tumor CD4IL2RALO Tregs to normal tissue CD4IL2RALO Tregs for possible changes of the above‐referenced key pathways we identified in our studies. The esophageal tumor CD4IL2RALO Tregs showed significant upregulation of genes for mitochondrial Complexes I, III, and IV; OXPHOS; and glycolysis when compared with the CD4IL2RALO Tregs from normal esophageal tissue (**Figure** **14**). The esophageal tumor CD4IL2RALO Tregs showed significant down‐regulation of genes for apoptosis when compared to the CD4IL2RALO Tregs from normal esophageal tissue (Figure 14). These findings, combined with our results on the effect of Mito‐ATO treatment, suggest that Mito‐ATO metabolically reprograms key pathways in the tumor CD4IL2RALO Tregs to patterns that promote their apoptosis. A recent study showed that Mito‐ATO inhibits Complex I and III activity in cancer cells,^[^ ^1^ ^]^ and another study revealed that Complex III is essential for the immunosuppressive function of Tregs.^[^ ^21^ ^]^ Our results are consistent with these studies and provide new clues by showing that Mito‐ATO significantly inhibits gene expression in a manner that would suppress energy generation and promote apoptosis in CD4IL2RALO Tregs within tumors. Because CD4IL2RALO cells significantly block cytotoxic CD4^+^ T cells from killing tumor cells,^[^ ^18^ ^]^ the strong inhibition of CD4IL2RALO Tregs by Mito‐ATO could contribute to the observed increased proportion of activated cytotoxic CD 4^+^ T cells in TIME (Figures 10D and 11C).

## 3 Discussion

Mitochondria have fundamental roles in maintaining homeostasis of the immune system via regulating metabolic pathways through glucose oxidation as well as the biosynthesis of fatty acids, amino acids, and hormones.^[^ ^7b^ ^]^ The regulation of metabolism in immune cell subsets is important for their survival, proliferation, and activation.^[^ ^22^ ^]^ Increasing evidence suggests that cellular metabolism is a critical determinant of the viability and function of both cancer cells and immune cells.^[^ ^7^, ^23^ ^]^ It is believed that altered metabolism in cancer cells prevents immune attack by creating an effective immunosuppressive TIME.^[^ ^7b^ ^]^ Here, we showed that Mito‐ATO inhibits tumor growth in both transplanted tumor models and in a spontaneous arising tumor model. Remarkably, in situ vaccination with Mito‐ATO significantly prevented lung cancer metastases from growing in the brain. Analyses of the potential effects of Mito‐ATO on immune cells in the TIME showed that Mito‐ATO treatment significantly reduces both G‐MDSCs and Tregs, presumably by targeting their cellular and mitochondrial metabolism. For G‐MDSCs, Mito‐ATO inhibits gene expression for mitochondrial complex I, complex V, OXPHOS, and glycolysis, which is associated with apoptosis of i.t. G‐MDSCs. For Tregs, Mito‐ATO inhibits gene expression for mitochondrial complex I, III, and IV; OXPHOS; and glycolysis, leading to elevated apoptosis of i.t. CD4IL2RALO Tregs. Reduction of i.t. G‐MDSCs and Tregs may facilitate the observed increase of tumor‐infiltrating cytotoxic CD4^+^ T cells inside the tumors. Interestingly, Mito‐ATO is able to enhance the efficacy of immune‐checkpoint blockade with anti‐PD‐1 in mice bearing immune checkpoint inhibitor‐resistant B16F10 melanoma. Our data suggest that this enhanced antitumor efficacy is a consequence of reduced G‐MDSCs and Tregs within the TIME by Mito‐ATO, and a concomitant increase in functional tumor‐infiltrating cytotoxic CD4^+^ T cells. Given their prominent roles in tumor immune evasion, targeting G‐MDSCs and Tregs with Mito‐ATO could be an attractive approach to modulate tumor immunity to prevent and treat cancers.

scRNA‐seq showed that Mito‐ATO suppresses the expression of genes for mitochondrial complex I, complex V, OXPHOS, and glycolysis and induces genes that should promote the death of G‐MDSCs in the TIME. Previous studies have shown that increased infiltration of G‐MDSCs in tumors is associated with poorer patient outcomes.^[^ ^24^ ^]^ Tumor‐associated MDSCs appear to be highly dependent on aerobic glycolysis and OXPHOS for ATP production compared with MDSCs in the periphery.^[^ ^25^ ^]^ G‐MDSCs from the spleens of tumor‐bearing mice showed increases in both aerobic glycolysis and OXPHOS compared with splenic neutrophils from the same mice in mouse breast cancer models, and 2‐DG, a glycolysis inhibitor, can suppress G‐MDSCs in the TIME.^[^ ^26^ ^]^ Similarly, we found that gene expression profiles for glycolysis, mitochondrial complex assemblies, and metabolic pathways were significantly altered in the G‐MDSCs from tumors compared with normal tissues in mice. Specifically, genes supporting glycolysis and mitochondrial electron complexes I, IV, and V in tumor G‐MDSCs were significantly higher than the G‐MDSCs in normal tissue. These results suggest that upregulation of glycolysis and OXPHOS in G‐MDSCs contributes to cell survival of MDSCs in the TIME in mice.^[^ ^26^ ^]^ Blockade of glycolysis in tumor MDSCs can rapidly induce proapoptotic genes, which suggests that tumor MDSCs rely on high rates of glycolysis to enhance their accumulation in the TIME.^[^ ^26^ ^]^ The current study showed that Mito‐ATO inhibits the expression of genes for mitochondrial complexes I and V, OXPHOS, and glycolysis, leading to decreases in i.t. G‐MDSCs. We addressed whether the metabolic changes observed in G‐MDSCs are directly mediated by Mito‐ATO or are a consequence of reduced tumor growth. A prior report suggests that a mitochondrial‐targeting compound has the potential to accumulate in the MDSCs (and tumor cells).^[^ ^27^ ^]^ Our scRNAseq data showed that that the apoptosis pathway was activated upon Mito‐ATO treatment (Figure 4E). These findings are further supported by the flow cytometry analyses that Mito‐ATO induces late apoptotic cell death in G‐MDSCs in vivo in our flow study. These results indicate that Mito‐ATO decreases the proportion of G‐MDSCs by inducing their apoptotic death. Hence, metabolic disruption of the G‐MDSCs leads to their apoptosis and is therefore more likely a cause rather than a consequence of the reduced tumor growth.

In addition to their roles in the maintenance of immune self‐tolerance and homeostasis, Tregs are a distinct subset of CD4^+^ T cells and are ubiquitously present in the TIME where they promote tumor development and progression by dampening antitumor immune responses.^[^ ^28^ ^]^ Tregs have a unique metabolic profile characterized by a preferential reliance on the tricarboxylic cycle and mitochondrial respiration relative to CD4^+^ effector T cells.^[^ ^29^ ^]^ In addition, the Treg‐defining transcription factor, Foxp3, has been shown to promote mitochondrial respiration.^[^ ^30^ ^]^ Highly active Tregs or effector Tregs rely on the upregulation of glycolysis for optimal function.^[^ ^31^ ^]^ We saw that gene expression profiles for glycolysis, mitochondrial complex assemblies, and metabolic pathways were significantly altered in the esophageal tumor Tregs compared with Tregs in normal esophageal tissues of mice. Thus, in TIMEs with low glucose and high lactate, the metabolic reprogramming of Tregs allows them to resist lactate‐induced functional and proliferative suppression (unlike CD4^+^ effector T cells).^[^ ^30a^ ^]^ A recent study showed that mitochondrial complex III is essential for the immunosuppressive function of Tregs.^[^ ^21^ ^]^ Treg cell‐specific ablation of mitochondrial complex III results in the development of a fatal inflammatory disease early in life, and mice lacking mitochondrial complex III specifically in the Tregs display a loss of the immunosuppressive capacity of Tregs.^[^ ^24^ ^]^ Compared with effector CD4^+^ T cells, Tregs depend more on mitochondrial respiration for energy requirements.^[^ ^21^ ^]^ Mitochondrial complex III knockout mice show decreased mitochondrial respiration and increased glycolytic metabolism.^[^ ^21^ ^]^ Mice with a conditional knockout of a complex III subunit fail to develop tumors using the B16 melanoma syngeneic mouse model, indicating the importance of mitochondrial respiration to the immunosuppressive function of Tregs in vivo.^[^ ^21^ ^]^ Interestingly, we showed that Mito‐ATO significantly inhibited mitochondrial complexes III, I, and IV in a functional subtype of Tregs. Specifically, we identified a specific Treg state, CD4IL2RALO, that could be specifically inhibited by Mito‐ATO treatment. This is the first time that a CD4IL2RALO subset has been identified in mice using scRNA‐seq. Both types of Tregs (IL2RAHI Treg and IL2RALO Treg) have suppressive effects on the cytotoxic CD4^+^ T cells’ antitumor immunity, with IL2RALO Tregs being less immunosuppressive than the IL2RAHI Tregs.^[^ ^32^ ^]^ Although there was an increase in the IL2RAHI Tregs percentage, we found the total Tregs percentage within the total CD4+ T cells were decreased after Mito‐ATO treatment. It was not clear as to why IL2RAHI cells are increased by Mito‐ATO or the importance of the IL2RALO Treg population underlying Mito‐ATO's antitumor effect, both of which warrant further investigation in the future. Interestingly, our results suggest that Mito‐ATO can downregulate the mitochondrial electron complex assembly and the main energy metabolism pathways while promoting apoptosis of the CD4IL2RALO Tregs. Therefore, it is plausible that the ability of Mito‐ATO treatment to complement PD‐1 immune checkpoint therapy results from its ability to suppress CD4IL2RALO Tregs, the subclass that is more resistant to anti‐PD‐1 alone. This premise is consistent with our results in Figure 4.

Our data show that in situ vaccination with Mito‐ATO elicits local and systemic antitumor immunity and stimulates immune‐mediated responses in untreated tumors. Careful examination of the TIME in Mito‐ATO treated tumors showed decreased i.t. G‐MDSCs and Tregs and increased CD4^+^ effector T cells. We also noticed an increase in the cytotoxic CD4^+^ T cells, but no changes in the G‐MDSCs or Tregs in the TIME of the nontreated side tumor. Mito‐ATO appears to inhibit OXPHOS (by suppressing gene expression for mitochondrial complexes) and to suppress the expression of genes for glycolysis; these changes may promote the cell death of G‐MDSCs and Tregs in the TIME. By analyzing the signature genesets of the OXPHOS pathway and complex I to complex V assemblies for all immune cell populations using the Seurat program, G‐MDSCs and CD4IL2RALO Tregs are clearly the major immune cell populations targeted by Mito‐ATO treatment. Both G‐MDSCs and CD4IL2RALO Tregs showed reduced OXPHOS activity and the activity of mitochondrial complexes (Complexes I, III, IV for CD4IL2RALO Tregs and Complexes I and V for G‐MDSCs). By contrast, in all other immune cells examined from Mito‐ATO‐treated tumors, no significant changes in mitochondrial metabolic pathways were observed. One possible mechanism for preferentially targeting the G‐MDSCs and CD4IL2RALO Tregs by Mito‐ATO could be due to the differential mitochondrial membrane potentials among the immune cells in the TIME.^[^ ^1^, ^7^, ^33^, ^34^ ^]^ A future comprehensive assessment of mitochondrial membrane potentials among the immune cells in the TIME is required to fully address this question. Overall, our findings show a selective, inhibitory effect of Mito‐ATO on immune suppression driven by G‐MDSCs and/or Tregs and support Mito‐ATO as a potent immunomodulator for cancer immunoprevention and immunotherapy.

We also found that Mito‐ATO could enhance the antitumor activity of PD‐1 blockade in a resistant B16F10 mouse melanoma model. Treatment with anti‐PD‐1 antibody alone only slightly inhibited tumor growth of B16F10 melanoma cells, while a relatively low dose of Mito‐ATO (2 × 10^−3^ m) alone inhibited but did not eradicate tumor growth. However, the combination of both anti‐PD‐1 antibody and Mito‐ATO significantly inhibited the growth of B16F10 melanoma tumors with complete eradication of seven of eight treated melanoma tumors. These findings are consistent with the selective inhibitory effect of Mito‐ATO on G‐MDSCs and Tregs within tumors, which will decrease immune suppression and thereby improve the antitumor activity of PD‐1 blockade. The data warrant future experiments on the effects of Mito‐ATO/PD‐1 blockade in other solid tumor models and examination of the combination of Mito‐ATO with other immune checkpoint inhibitors including CTLA‐4 or VISTA. Our findings show a selective, inhibitory effect of Mito‐ATO on immune suppression driven by G‐MDSCs and Tregs and that Mito‐ATO improves the antitumor activity of PD‐1 blockade. These results provide a rational basis for future clinical evaluation of combined immunotherapy using Mito‐ATO plus PD‐1 blockade.

## 4 Experimental Section

### Synthesis and Characterization of Mito‐ATO

Mito‐ATO was synthesized according to previously published methods,^[^ ^1^ ^]^ and its purity (>95%) was verified by high‐performance liquid chromatography. Stock aliquots of Mito_10_‐ATO (which is defined by having a 10‐carbon linker between the TPP^+^ and ATO, and hereafter referred as Mito‐ATO) were dissolved in dimethyl sulfoxide (DMSO) and then further diluted with PBS (1:3 v/v) for use in in vivo experiments. The vehicle control was prepared as DMSO/PBS (1:3 v/v).

### Cell Lines and Animals

LKR13 cells, a mouse lung adenocarcinoma line that expresses mutant KrasG12D on the SV129 background, were a generous gift from Dr. Jonathan M. Kurie (MD Anderson).^[^ ^35^ ^]^ LKR13‐luc cells were generated by transfecting LKR13 cells with CMV‐firefly luciferase lentivirus (Cellomics Technology, PLV‐10064‐200). UN‐SCC680 cells, a mouse squamous carcinoma cell line on the A/J mouse background, were a generous gift from Dr. Luis M. Montuenga (University of Navarra).^[^ ^36^ ^]^ LLC cells were obtained from ATCC (#CLR‐1642) (on the C57BL/6J mouse background) and were maintained in RPMI 1640 medium (Thermo Fisher Scientific, #11875) supplemented with 10% fetal bovine serum. LKR13 cells and UN‐SCC680 cells were cultured in complete media consisting of RPMI‐1640 (Thermofisher, 11875‐093) supplemented with 10% fetal bovine serum (Sigma, F4135) and 1% penicillin/streptomycin (Gibco,15140‐122). LKR13‐luc cells were cultured in complete media supplemented with geneticin (500 µg mL^−1^) for selection of neomycin‐resistant cell lines, indicating maintenance of the transfected luciferase gene.

Six‐ to seven‐week‐old wild type female sv129 mice and A/J mice were purchased from the Jackson Laboratory. Mice were acclimatized one week following arrival to the facility. Transgenic C3 (1)/Tag mice were a generous gift from Dr. Jeffrey E. Green (National Cancer Institute, National Institutes of Health).^[^ ^8^ ^]^ FVB/N wild‐type mice were purchased from the Jackson Laboratory. For all experiments, only the F2 C3(1)/SV40‐T/t‐antigen (C3(1)/Tag) generation of mice were used. Mice were kept in the Biomedical Resource Center at the Medical College of Wisconsin, Milwaukee, WI, and all procedures were approved by the Institutional Animal Care and Use Committee. 

### Mouse Models and Tumor Treatment Studies

In the dual tumor models, LKR13 (2 × 10^6^) or UN‐SCC680 (5 × 10^6^) tumor cells were subcutaneously inoculated on the left and right sides of the mouse's abdomen. When tumors reached an average size of 100 mm^3^ (usually seven to nine days after tumor inoculation), mice were randomized into treatment groups. 50 µL of Mito‐ATO (5 × 10^−3^ m) or vehicle was administered by i.t. injection into the right‐side tumor every other day for a total of four (UN‐SCC680) or five (LKR13 cells) treatments. Tumor sizes were measured every four days for both the left‐ and right‐side tumors using digital calipers (World Precision Instruments, 501601). 

In the distant‐side tumor rechallenge models, LKR13 (2 × 10^6^) or UN‐SCC680 (5 × 10^6^) tumor cells were subcutaneously inoculated only onto the right‐side of the mouse abdomen. Mice received the same treatment methods (by injection into the tumor) according to the descriptions in the dual tumor model. Eight days after the right‐side treated tumor had disappeared, the left‐side abdomen of each mouse or their age‐matched naïve counterpart was subcutaneously inoculated with LKR13 (1 × 10^6^) or UN‐SCC680 (2 × 10^6^) tumor cells and observed for tumor development.

For the lung cancer brain metastasis study, six‐week‐old female SV129 mice (previously cured of LKR13 lung carcinoma via i.t. injection with Mito‐ATO) were used. Ten days after tumor regression, 5 × 10^5^ LKR13‐luc tumor cells in 100 µL of PBS were injected into the left ventricle of the cured mice and their age‐matched littermates under ultrasound guidance (Vevo 3100, FUJIFILM Visual Sonics). Brain metastases were monitored periodically by bioluminescence using a Xenogen IVIS‐200 system (Alameda, CA). The survival rate was monitored on a daily basis. Upon euthanization, metastases were confirmed with ex vivo luminescence and histopathology.

In the C3(1)/Tag transgenic mammary tumor mouse model, when the first tumor reached an average size of 80 mm^3^, i.t. injections of Mito‐ATO or vehicle i.t. were started every other day for a total of three injections. Development of the treated and nontreated tumors in the same animal was monitored. Both tumor burden and tumor numbers were recorded. Body weights were monitored every week.

To deliver Mito‐ATO systemically by oral gavage, LLC‐Luc cells (2 × 10^5^) cells were injected either orthotopically or into the left ventricle. One day after the inoculation, C57BL/6J were given daily oral gavage of Mito‐ATO at a dose of 600 µg (or 30 mg kg^−1^ body weight). Quantitative data for bioluminescence imaging of the orthotopic growth (*n* = 8 per group) or brain metastasis (*n* = 7 per group) of LLC‐Luc cells were collected.

In experiments where Mito‐ATO was combined with anti‐PD‐1 treatment, five‐week‐old female C57BL/6J mice were purchased from Jackson Laboratory. Mice were inoculated with 5 × 10^5^ B16F10 cells on the right‐side flank. On day six postcell inoculation, mice were randomized into different treatment groups: (a) intraperitoneal (i.p.) injection with PBS and i.t. injection with vehicle (25% DMSO/PBS), (b) i.p. injection with anti‐PD1 (Bioxcell, BE0146) and i.t. injection with vehicle, (c) i.p. injection with PBS and i.t. injection with Mito‐ATO (2 × 10^−3^ m), (d) i.p. injection with anti‐PD1 and i.t. injection with Mito‐ATO (2 × 10^−3^ m). Mice started treatments on day 7 and tumor sizes were measured every two days. Mice were followed until death or were euthanized earlier if tumors reached 2000 mm^3^. 

### Depletion of CD4 and CD8 T Cells

Anti‐CD4 (GK1.5 clone‐ rat IgG2b, 250 µg, BioXcell, BP0003‐1) or anti‐CD8 mAbs (2.43 clone‐rat IgG2b, 250 µg, BioXcell, BP0061) were injected i.p. one day before and one day after tumor inoculation followed by repeat injections once per week. Eight days after the first i.p. injection, the spleens of the mice were collected to verify the depletion of CD4^+^ and CD8^+^ T cells using flow cytometry. The results showed greater than 99% depletion of each cell subset. 

### Flow Cytometry

Forty‐eight hours after the second i.t. administration of Mito‐ATO, both the treated and nontreated tumors were harvested. Tumors were minced into 2 mm^3^ pieces and digested with mouse tumor dissociation buffer (Miltenyi Biotec, CA, 130‐096‐730) at 37 °C for 30 min and passed through a 40 µm nylon mesh to generate single cell suspensions per the manufacturer's instructions. Red blood cells were removed by red blood cell lysis buffer (155 × 10^−3^ m NH_4_Cl, 10 × 10^−3^ m KHCO_3_, 0.1 × 10^−3^ m EDTA). Isolated cells were first stained for viability and cell surface markers. Violet fluorescent reactive dye (Invitrogen, MP34955) was used to identify viable cells. Antibodies for staining surface markers included BV786 anti‐CD45 (Clone: 30‐F11), PE anti‐CD3 (Clone: 17A2), FITC anti‐CD4 (Clone: GK1.5), BUV396 anti‐CD8a (Clone: 53‐6.7), FITC anti‐CD11b (Clone: M1/70), APC/Fire750 anti‐F4/80 (Clone: BM8), BUV396 anti‐Ly6G (Clone: 1A8), PE/Cy7 anti‐ Ly6C (Clone: HK1.4), and APC/Fire750 anti‐CD25 (Clone: PC61). For transcription factor staining, cells were first stained with surface markers, then fixed with fixation buffer (Biolegend, 420801), permeabilized with FoxP3/Transcription Factor Staining Buffer Set (eBioscience, 00‐5523‐00), and stained with APC anti‐FoxP3 (Clone: FJK‐16s). For intracellular cytokine staining, cells were stimulated for 4 h at 37 °C in Roswell Park Memorial Institute medium containing 10% fetal bovine serum, 2 × 10^−3^ m l‐glutamine, 50 × 10^−6^ m 2‐mercaptoethanol, 1% penicillin–streptomycin, 0.2% of cell stimulation cocktail (eBioscience, 00‐4970‐93), 0.1% of monensin (eBioscience, 00‐4505‐51), and Brefeldin A (eBioscience, 00‐4506‐51). Cells were then surface‐stained with antibodies, fixed, and permeabilized using FoxP3/transcription factor staining buffer set, stained with intracellular cytokine staining buffer containing PE anti‐IFN‐*γ* and PE‐Cy7 anti‐TNF‐*α* antibodies, and then analyzed by flow cytometry. Cells incubated in media lacking PMA/ionomycin served as nonstimulated controls. To analyze myeloid‐derived cells, cells were additionally incubated with anti‐Mo CD16/CD32 (Invitrogen, 14‐0161‐82). These flow cytometry antibodies were purchased from either Biolegend, eBioscience, or BD Biosciences. Cells were analyzed using an LSR Fortessa X‐20 flow cytometer (Becton Dickinson). Data were analyzed using FlowJo software (Treestar, Inc.).

### Apoptosis Measurement

To analyze levels of apoptosis induced by Mito‐ATO in vivo, seven‐week‐old SV129 mice were inoculated with LKR13 (2 × 10^6^) tumor cells on the right abdomen. Eight days postinoculation, Mito‐ATO (5 × 10^−3^ m) was i.t. injected into tumor. Forty‐eight hours later, mice were euthanized, and tumors were incised and dissociated into single cells suspension. Cells were first stained with cell‐type‐specific phenotypic and apoptotic markers and subjected to flow cytometric analysis. The levels of cell apoptosis were determined by FlowJo software. Early and late apoptotic cells were defined as Annexin V+PI− and Annexin V+PI+, respectively. A minimum of 20 000 events per sample was acquired.

### Detection of Mito‐ATO Concentration in Plasma and Tumor Using LC‐MS

Six‐ to seven‐week‐old wild type female sv129 mice were purchased from the Jackson Laboratory. Mice were acclimatized one week following arrival to the facility. LKR13 (2 × 10^6^) tumor cells were subcutaneously inoculated on the left and right sides of each mouse's abdomen (*n* = 6). When tumors reached an average size of 100 mm^3^, 50 µL of Mito‐ATO (5 × 10^−3^ m) was administered by i.t. injection into the right‐side tumor. Blood samples were collected from the submandibular vein 1 h post i.t. injection into the EDTA‐containing tube. Blood samples were centrifuged at 2500 × *g* for 15 min, and the plasma was stored at −80c until analysis. Tumor samples were collected 1 h post i.t. injection, weighed, homogenized in fourfold volume methonal/water (50/50 v/v) and stored at −80c until analysis. For plasma, 20 µL of mouse plasma samples were added to 80 µL of methanol containing internal standard (5 × 10^−6^ m Mito‐HNK), vortexed, and spun at 15 000 × *g* for 15 min at 4 °C. 60 µL of supernatant was injected into the UHPLC‐QQQ for analysis. To quantify Mito‐ATO in the plasma samples, methanol stock solutions with different concentrations of Mito‐ATO were spiked into blank mouse plasma, and the calibration samples were prepared in the same way as actual samples. The linear range of the calibration curve for plasma samples was 0.01 × 10^−6^– 5 × 10^−6^ m with good linearity (R^2^ = 0.9844, weight 1/x). For tumor samples, 20 µL of mouse tumor homogenate samples were added to 160 µL of methanol containing internal standard (5 × 10^−6^ m Mito‐HNK), vortexed, and spun twice at 15 000 × *g* for 15 min at 4 °C. Samples from the treatment group were diluted 250‐fold with blank tumor homogenate before the internal standard solution was added. 100 µL of supernatant was injected into the UHPLC‐QQQ for analysis. To quantify Mito‐ATO in the tumor homogenate samples, methanol stock solutions with different concentrations of Mito‐ATO were spiked into blank mouse tumor homogenate, and the calibration samples were prepared in the same way as actual samples. The linear range of the calibration curve for tumor samples was 0.02 × 10^−6^– 5 × 10^−6^ m with good linearity (R^2^ = 0.9891, weight 1/x2). The plasma and tumor samples were analyzed using a Thermo TSQ Quantis coupled with a Thermo Vanish UHPLC. The analyte and the internal standard were separated on a Phenomenex Luna Omega C18 column (1.0 mm × 50 mm, 1.6 µm), and eluted by a water‐acetonitrile mobile phase system (both containing 0.1% formic acid). The flow rate was set at 0.15 mL min^−1^, and the gradient was as follows: 0–0.2 min, 50% B; 0.2–1.2 min, 50–98% B; 1.2–4 min, 98% B; 4–4.3 min, 98–50% B; 4.3–5 min, 50% B. The retention times of Mito‐ATO and Mito‐HNK were 2.42 and 2.16 min, respectively. The column temperature was 40 °C, and the injection volume was 2 µL. Mito‐ATO and Mito‐HNK were monitored under the selected reaction monitoring mode coupled with a positive electrospray ionization source. The quantification ion transition was 767.48→401.25 for Mito‐ATO (collision energy 51.42 eV), and 667.51→262.14 for Mito‐HNK (collision energy 48.81 eV). The ion spray voltage was 3500 V. High‐purity nitrogen was used as the sheath gas (50 arbitrary unit), aux gas (10 arbitrary unit), sweep gas (1.0 arbitrary unit), and collision gas. The temperatures of the ion transfer tube and vaporizer were 325 and 350 °C, respectively. Signals from 1 to 4 min were recorded by the mass spectrometer.

### scRNA‐seq

Forty‐eight hours after the second administration of i.t. Mito‐ATO, both treated and nontreated tumors were harvested. Tumors were processed into single cell suspensions using the methods described in the Flow Cytometry section. Cells were directly stained with violet viability dye and APC anti‐CD45 for 30 min. CD45^+^ cells were flow sorted (BD FACSAria II Cell sorter), and the sorted cells were centrifuged at 500 × *g* for 5 min, and counted manually with a Neubauer Chamber. Approximately 1.6 × 10^4^ cells were loaded onto the 10× Chromium Controller per the manufacturer's instructions, resulting in recovery of about 1.0 × 10^4^ cells. The scRNA‐seq libraries were generated by Chromium Single Cell 3′ v3 Reagent Kits (10× Genomics) and sequenced using NextSeq 500/550 High Output Kits v2 (150 cycles) (Illumina) according to the manufacturer's protocol. There were two replicates for each experimental group. The standard quality control procedures were followed to filter out the dead cells or unhealthy dying cells according to the widely used procedures practiced in the single‐cell RNA‐seq research field (the related publication can be found in the following github website (https://github.com/hbctraining/scRNA‐seq_online/blob/master/lessons/04_SC_quality_ control.md)). In general, UMI (Unique Molecular Identifier) counts per cell should be above 500. Here, more stringent filtering criteria were used. The range of UMI counts for the G‐MDSC cell population in the studies ranged from 1572 to 28 256. The mean of UMI counts for the G‐MDSC population in the studies was 8328. These data indicate that for the G‐MDSCs the general quality control filtering criteria were exceeded.

Raw sequencing data were demultiplexed and converted to gene‐barcode matrices using the Cell Ranger (version 2.2.0) mkfastq and count functions, respectively (10× Genomics). The mouse reference genome mm10 was used for alignment. Data were further analyzed in R (version 3.4.0) using Seurat (version 3). The number of genes detected per cell, the number of unique molecular identifiers, the percent of mitochondrial genes were plotted, and outliers were removed (cells that expressed less than 200 and more than 2500 genes) to filter out doublets (two single cells) and dead cells. Differences in the number of unique molecular identifiers and percent of mitochondrial reads were regressed out. Raw unique molecular identifier counts were normalized and log transformed. To analyze the sequenced CD45^+^ cells from mouse lung tumors, the Seurat R package3 was utilized to perform fine clustering of the single cells.^[^ ^37^ ^]^ The gene expression data from all single cells were aligned and projected in a 2D space through uniform manifold approximation and projection to allow identification of the cell populations among the CD45^+^ cells.

Differential gene expression analysis of scRNA‐seq data was performed as follows: Before the differential expression analysis, the computational imputation of zero values was performed to correct for the influence of dropout events (i.e., failure in detecting expressed genes due to low sequencing depth of single cells). Computational methods described previously were utilized^[^ ^38^ ^]^ to perform imputation and other data processing procedures. Specifically, gene expression levels were quantified using metric log2 (TPM+1). Transcripts per million is a normalization method for RNA‐seq and should be read as “for every 1 000 000 RNA molecules in the RNA‐seq sample, *x* came from this gene/transcript.” Missing gene expression values were imputed using the scImpute algorithm with default parameters and transcripts per million values and gene lengths (for a gene associated with multiple transcripts, the length of the longest transcript was used) as the input. Imputation was only applied to genes with dropout rates (i.e., the fraction of cells in which the corresponding gene has zero expression value) larger than 50% to avoid over‐imputation. The imputated scRNA‐seq data were then subjected to differential expression analysis using the DEsingle program to assess differences between the control and Mito‐ATO treatment groups. A list of the overall differential expression results was used as input into the GSEAPreranked tool implemented in the GSEA (Gene Set Enrichment Analysis) program. Hallmark genesets listed in the MSigDB (molecular signatures database: https://www.gsea‐msigdb.org/gsea/msigdb/index.jsp) were used to test gene expression signatures to detect the important biological processes that were affected by Mito‐ATO treatment. These analyses may provide insights into the mechanism(s) underlying the efficacy of Mito‐ATO treatment in preventing or treating cancer.

For the pathway activity analyses, different immune cell populations were identified using the Seurat program.^[^ ^37^, ^39^ ^]^ The signature genesets of the OXPHOS pathway and complex I to complex V assemblies were downloaded from the MSigDB‐GSEA database (http://www.gsea‐msigdb.org/gsea/msigdb/index.jsp). The set of biological pathway genes overexpressed in the low mitochondrial membrane potential mouse cells was determined according to recent research.^[^ ^7a^ ^]^ Specifically, this gene expression signature consists of the following genes: *Zmat3*, *Ccng1*, *Mdm2*, *Tnfrsf10b*, *Ccng1*, *Rb1*, *Nfyb*, *Lhx2*; higher (upregulated) signature scores of these genes denote low‐Δ*ψ*_m._ When scoring cells for the expression of the above known gene signatures, the AddModuleScore function was used in Seurat in the same manner as done in the other scRNA‐seq studies.^[^ ^33^, ^37^ ^]^ The comparison of the signature scores of the cells of each immune population between the Mito‐ATO treated and untreated mouse lung tumors was performed using the t_test and cohens d functions implemented in the rstatix R package (https://rpkgs.datanovia.com/rstatix/). The dotplot of the regulation of mitochondrial metabolic pathways activity by Mito‐ATO treatment compared with the untreated control samples across different immune cell populations within the mouse lung TIME was made by using the ggplot2 R package (https://www.rdocumentation.org/packages/ggplot2/versions/3.3.3).

### Statistical Analysis

General statistical analyses were performed using GraphPad Prism 7.0. An unpaired, two‐tailed Student's *t*‐test, or one‐way or two‐way analysis of variance were used for column, multiple columns, and group analyses, respectively. ∗ *P* < 0.05, ∗∗ *P* < 0.01, ∗∗∗ *P* < 0.001, and ∗∗∗∗ *P* < 0.0001 were considered statistically significant.

## Conflict of Interest

M.Y. is a co‐founder of OncoC4, Inc. All other authors declare no conflict of interest.

## Data Availability

The data that support the findings of this study are available from the corresponding author upon reasonable request.
